# Supplementary material for: Healthcare resource utilization in patients with treatment-resistant depression—A Danish national registry study
Source: PLoS One. 2022 Sep 27;17(9):e0275299. doi: 10.1371/journal.pone.0275299 (PMC9514626; doi:10.1371/journal.pone.0275299)
Supplement: S1 File — (DOCX) [file pone.0275299.s001.docx]

**Supporting Information S1 File**

Supplementary material to the manuscript ‘Healthcare resource utilization in patients with treatment-resistant depression – a Danish national cohort study’

S1 Figure. Study design, observation periods and definition of index date

S2 Figure. Somatic healthcare resource utilization by depression severity

S3 Figure. Somatic healthcare resource utilization by year of MDD diagnosis

S4 Figure. Time trends of total medicine sales to adults^1^ in the primary sector in Denmark

S1 Table. Fixed prices of healthcare services

S2 Table. Average healthcare resource utilization costs (EUR) in the year before and after the index date

S3 Table. Average healthcare resource utilization costs (EUR) in the year before and after the index date, stratified by depression severity

S4 Table. Average healthcare resource utilization costs (EUR) in the year before and after the index date, stratified by year of MDD diagnosis

S5 Table. Average healthcare resource utilization comparing TRD patients with non-TRD patients in the matched population, abstaining from restriction to patients being alive 2 years after MDD index

**S1 Figure. Study design, observation periods and definition of index date**


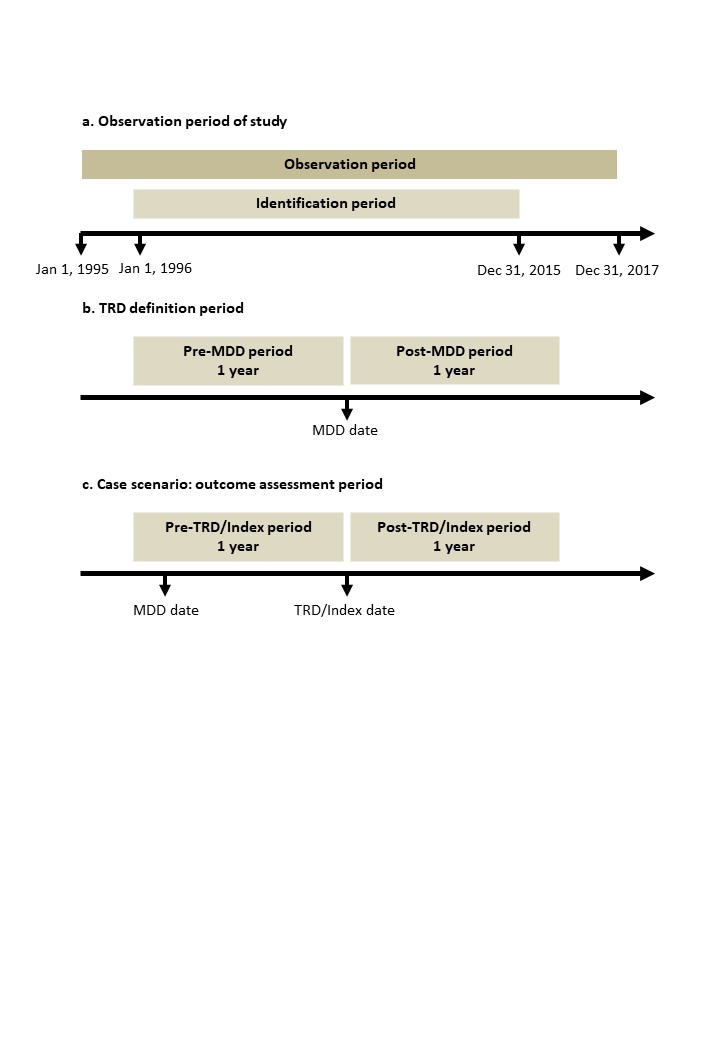


**S2 Figure. Somatic healthcare resource utilization by depression severity**

^1^ The fraction of users of the given somatic healthcare service for TRD patients and non-TRD patients of the match population in the year following the index date.

^2^ The relative risk (RR) estimate comparing TRD with non-TRD patients in each stratum is presented with 95% confidence interval (CI) and P-value.

^3^ Vertical line represents RR=1.

**S3** **Figure. Somatic healthcare resource utilization by year of MDD diagnosis**

^1^ The fraction of users of the given psychiatric healthcare service for TRD patients and non-TRD patients of the match population in the year following the index date.

^2^ The relative risk (RR) estimate comparing TRD with non-TRD patients in each stratum is presented with 95% confidence interval (CI) and P-value.

^3^ Vertical line represents RR=1.

**S4 Figure.** **Time trends of total medicine sales to adults^1^ in the primary sector in Denmark**

Source: medstat.dk, accessed March 28, 2020.

^1^ Defined as ≥18 years of age

^2^ Antidepressive medicine (ATC code N06A)

^3^ Other non-antidepressive psychiatric medicine (ATC codes N05A, N05B)

**S1 Table. Fixed prices of healthcare services**

| **Type of HRU** | **Price** | **Source** |
| --- | --- | --- |
| Psychiatric outpatient visit and home visit | 1,874 DKr  (€251) | “Psykiatritakster 2019 (Excel)”, <https://sundhedsdatastyrelsen.dk/da/afregning-og-finansiering/takster-drg/takster-2019> |
| Somatic outpatient visit | 1,958 DKr  (€262) | “DRG takster 2019 (Excel)”, code 19MA98, <https://sundhedsdatastyrelsen.dk/da/afregning-og-finansiering/takster-drg/takster-2019> |
| Psychiatric emergency room visit | 1,874 DKr  (€251) | Assumed same as psychiatric outpatient visit |
| Somatic emergency room visit | 1,958 DKr  (€262) | Assumed the same as somatic outpatient visit |
| Acute/elective somatic hospital bed day | 17,295 DKr (day 1-4) (€2315) and  2,077 DKr/day  (€278) after day 4 | “DRG takster 2019 (Excel)”, code 19MA02, https://sundhedsdatastyrelsen.dk/da/afregning-og-finansiering/takster-drg/takster-2019 |
| Acute/elective psychiatric hospital bed day | 3,745 DKr  (€501) | “Psykiatritakster 2019 (Excel)”, <https://sundhedsdatastyrelsen.dk/da/afregning-og-finansiering/takster-drg/takster-2019> |
| Private healthcare specialist (GP, psychologist, psychiatrist, and other private primary healthcare specialist) | Prices for private healthcare specialist services were retrieved from the National Health Insurance Service System using the “honorar” table. | |

**S2 Table. Average healthcare resource utilization costs (EUR) in the year before and after the index date**

|  | TRD | | Non-TRD | | TRD vs. non-TRD  (after index) | |
| --- | --- | --- | --- | --- | --- | --- |
| Variable^1^ | Before index  mean (sd)^2^ | After index  mean (sd)^2^ | Before index  mean (sd) | After index  mean (sd) | Change (%)  in risk of cost (95% CI)^3^ | Change (%)  in costs  (95% CI)^4^ |
| **Year 2006–2015** | |  |  |  |  |  |
| Total, excl. medicine | 11,997 (16,017) | 9,302 (14,873) | 8,071 (10,740) | 5,850 (11,289) | 0.6 (0.5; 0.7) | 108.7 (102.4; 115.2) |
| Total (DRG), excl. medicine | 13,611 (57,252) | 9,956 (26,912) | 10,257 (18,999) | 6,676 (22,749) | 0.7 (0.6; 0.8) | 105.5 (99.1; 112.2) |
| Total, incl. medicine | 12,588 (16,108) | 10,256 (15,068) | 8,551 (10,910) | 6,412 (11,488) | 0.5 (0.5; 0.6) | 107.5 (101.9; 113.1) |
| Total (DRG), incl. medicine | 14,201 (57,285) | 10,910 (27,018) | 10,736 (19,119) | 7,238 (22,875) | 0.5 (0.5; 0.6) | 105.6 (99.9; 111.4) |
| Psychiatric^5^ | 7,499 (13,793) | 6,323 (13,554) | 3,431 (7,730) | 2,823 (9,414) | 53.2 (51.3; 55.1) | 66.4 (60.9; 72.2) |
| Psychiatric (DRG)^5^ | 7,754 (55,173) | 6,217 (24,916) | 4,012 (11,818) | 2,727 (19,276) | 52.4 (50.5; 54.3) | 66.7 (61.1; 72.5) |
| Somatic | 4,091 (8,226) | 2,647 (5,846) | 4,308 (7,865) | 2,737 (6,205) | 2.3 (0.9; 3.7) | -2.0 (-5.1; 1.2) |
| Somatic (DRG) | 5,449 (15,297) | 3,407 (10,004) | 5,913 (15,353) | 3,659 (12,152) | 2.3 (0.8; 3.8) | -2.6 (-6.3; 1.2) |
| ^1^ Estimates restricted to the period (year of MDD diagnosis) between 2006 to 2015, whence Diagnosis Related Groups (DRG) prices were available. Estimates based on fixed prices or DRG prices.  ^2^ Mean healthcare utilization costs with standard deviation (sd) before and after the index date.  ^3^ The percentage change in risk of incurring any cost of the particular type of healthcare expenditure in the year after the index date is given for TRD relative to matched non-TRD patients, presented with 95% confidence interval (CI).  ^4^ The percentage change in costs in the year after the index date for TRD patients relative to matched non-TRD patients, presented with 95% confidence interval (CI).  ^5^ Psychiatric cost includes hospital-based psychiatric services, private psychiatrist services, private psychologist services and psychiatric home visits. | | | | | | |

**S3 Table. Average healthcare resource utilization costs (EUR) in the year before and after the index date, stratified by depression severity**

|  | TRD | | | Non-TRD | | | | TRD vs. non-TRD  (after index) | | | |  |
| --- | --- | --- | --- | --- | --- | --- | --- | --- | --- | --- | --- | --- |
| Variable | Before index  mean (SD) | After index  mean (sd) | | Before index  mean (sd) | | After index  mean (sd) | | Change (%) in risk of cost  (95% CI)^1^ | | Change (%) in costs (95% CI)^2^ | |  |
| **Year 1996–2015** |  |  | |  | |  | |  | |  | |  |
| Total, excl. medicine |  |  | |  | |  | |  | |  | |  |
| Mild | 11,829 (16,122) | 8,333 (14,064) | | 8,661 (11,708) | | 5,540 (10,642) | | 3.3 (2.7; 3.8) | | 80.8% (73.9%; 88.0%) | |  |
| Moderate | 11,894 (16,150) | 8,964 (15,580) | | 7,793 (11,475) | | 5,701 (11,927) | | 4.0 (3.4; 4.5) | | 91.7% (84.4%; 99.3%) | |  |
| Severe | 19,620 (21,851) | 12,427 (19,203) | | 12,408 (15,307) | | 8,724 (17,527) | | 3.4 (2.8; 4.1) | | 89.7% (79.6%; 100.3%) | |  |
| Total, incl. medicine |  |  | |  | |  | |  | |  | |  |
| Mild | 12,592 (16,239) | 9,459 (14,259) | | 9,270 (11,894) | | 6,229 (10,845) | | 1.3 (1.1; 1.4) | | 95.6% (89.0%; 102.4%) | |  |
| Moderate | 12,534 (16,238) | 10,010 (15,739) | | 8,279 (11,613) | | 6,306 (12,084) | | 1.2 (1.0; 1.3) | | 108.1% (101.0%; 115.4%) | |  |
| Severe | 20,177 (21,870) | 13,581 (19,382) | | 12,877 (15,400) | | 9,405 (17,676) | | 1.1 (0.9; 1.3) | | 102.7% (93.0%; 112.9%) | |  |
| Psychiatric |  |  | |  | |  | |  | |  | |  |
| Mild | 5,712 (12,652) | 4,802 (12,360) | | 2,114 (7,032) | | 1,711 (7,747) | | 91.2 (86.9; 95.5) | | 59.2% (51.4%; 67.5%) | |  |
| Moderate | 8,583 (14,911) | 6,499 (14,508) | | 4,246 (9,166) | | 3,216 (10,285) | | 45.8 (43.6; 48.0) | | 54.2% (47.9%; 60.9%) | |  |
| Severe | 16,431 (20,823) | 10,338 (18,532) | | 8,873 (13,902) | | 6,393 (16,594) | | 32.9 (30.8; 35.2) | | 52.1% (43.7%; 61.1%) | |  |
| Somatic |  |  | |  | |  | |  | |  | |  |
| Mild | 5,833 (10,262) | 3,271 (6,763) | | 6,322 (9,804) | | 3,605 (7,273) | | -0.6 (-2.2; 1.1) | | -4.4% (-8.1%; -0.5%) | |  |
| Moderate | 3,056 (6,161) | 2,253 (5,097) | | 3,340 (7,058) | | 2,300 (5,941) | | 3.5 (1.5; 5.6) | | -0.7% (-4.7%; 3.5%) | |  |
| Severe | 2,951 (6,101) | 1,892 (4,123) | | 3,335 (6,653) | | 2,154 (5,208) | | 3.7 (1.0; 6.5) | | -8.1% (-13.1%; -2.8%) | |  |
| GP |  |  | |  | |  | |  | |  | |  |
| Mild | 313 (224) | 247 (215) | | 235 (185) | | 195 (175) | | 1.3 (1.0; 1.7) | | 29.1% (25.5%; 32.7%) | |  |
| Moderate | 292 (204) | 215 (183) | | 233 (185) | | 177 (176) | | 1.3 (0.8; 1.7) | | 23.9% (20.5%; 27.5%) | |  |
| Severe | 280 (342) | 207 (200) | | 224 (169) | | 173 (163) | | 1.8 (1.2; 2.4) | | 21.1% (16.3%; 26.0%) | |  |
| Other private healthcare specialist | |  | |  | |  | |  | |  | |  |
| Mild | 128 (268) | 131 (323) | | 116 (276) | | 130 (361) | | 3.7 (2.0; 5.5) | | 6.0% (1.6%; 10.5%) | |  |
| Moderate | 99 (213) | 94 (233) | | 90 (212) | | 92 (264) | | 4.4 (2.6; 6.2) | | 2.5% (-1.5%; 6.6%) | |  |
| Severe | 83 (168) | 77 (192) | | 84 (210) | | 83 (228) | | 5.0 (2.3; 7.7) | | -6.0% (-11.0%; -0.7%) | |  |
| Medicine, other |  |  | |  | |  | |  | |  | |  |
| Mild | 433 (807) | 498 (913) | | 403 (812) | | 438 (831) | | 4.8 (3.9; 5.7) | | 20.1% (15.7%; 24.6%) | |  |
| Moderate | 319 (721) | 372 (775) | | 278 (652) | | 302 (738) | | 6.9 (5.8; 8.0) | | 26.9% (22.0%; 31.9%) | |  |
| Severe | 258 (589) | 311 (659) | | 237 (527) | | 267 (632) | | 7.7 (6.0; 9.3) | | 23.3% (16.7%; 30.3%) | |  |
| Medicine, other psychiatric | |  | |  | |  | |  | |  | |  |
| Mild | 45 (110) | 51 (143) | | 29 (84) | | 29 (109) | | 53.9 (50.1; 57.8) | | 25.0% (19.5%; 30.7%) | |  |
| Moderate | 39 (91) | 44 (117) | | 24 (67) | | 26 (105) | | 55.3 (51.4; 59.3) | | 26.4% (20.9%; 32.2%) | |  |
| Severe | 32 (69) | 41 (100) | | 24 (70) | | 25 (95) | | 53.3 (48.1; 58.7) | | 23.7% (16.6%; 31.2%) | |  |
| Medicine, antidepressant | |  | |  | |  | |  | |  | |  |
| Mild | 285 (388) | 576 (712) | | 177 (332) | | 222 (434) | | 37.8 (36.6; 39.0) | | 139.0% (131.1%;147.1%) | |  |
| Moderate | 283 (372) | 630 (715) | | 184 (334) | | 278 (475) | | 30.6 (29.6; 31.7) | | 124.6% (117.3%;132.2%) | |  |
| Severe | 267 (403) | 802 (934) | | 207 (387) | | 389 (656) | | 24.3 (23.1; 25.5) | | 118.4% (108.7%;128.5%) | |  |
| **Year 2006–2015^3^** |  | |  | |  | |  | |  | |  | |
| Total, excl. medicine |  | |  | |  | |  | |  | |  | |
| Mild | 11,036 (15,059) | | 8,373 (12,998) | | 8,091 (10,409) | | 5,352 (9,565) | | 0.6 (0.5; 0.8) | | 102.1% (92.5%; 112.2%) | |
| Moderate | 10,348 (14,064) | | 8,596 (14,181) | | 6,869 (9,784) | | 5,326 (10,281) | | 0.7 (0.5; 0.8) | | 110.1% (100.2%; 120.4%) | |
| Severe | 16,796 (19,739) | | 12,313 (18,524) | | 10,240 (12,530) | | 7,731 (15,141) | | 0.7 (0.4; 0.9) | | 118.8% (104.7%; 134.0%) | |
| Total (DRG) |  | |  | |  | |  | |  | |  | |
| Mild | 13,699 (23,809) | | 9,554 (24,383) | | 11,228 (19,441) | | 6,732 (15,016) | | 0.6 (0.5; 0.8) | | 97.0% (87.1%; 107.5%) | |
| Moderate | 12,515 (85,411) | | 9,460 (32,296) | | 8,464 (14,178) | | 5,761 (12,248) | | 0.7 (0.5; 0.8) | | 108.8% (98.6%; 119.5%) | |
| Severe | 15,458 (30,182) | | 11,607 (19,395) | | 11,752 (24,766) | | 8,250 (41,560) | | 0.7 (0.5; 0.9) | | 115.9% (101.7%; 131.1%) | |
| Psychiatric |  | |  | |  | |  | |  | |  | |
| Mild | 4,803 (10,890) | | 4,544 (10,866) | | 1,689 (5,407) | | 1,452 (6,261) | | 91.1 (86.0; 96.4) | | 68.9% (58.9%; 79.4%) | |
| Moderate | 6,944 (12,446) | | 6,037 (12,921) | | 3,411 (7,250) | | 2,837 (8,653) | | 42.7 (40.2; 45.2) | | 62.3% (54.3%; 70.8%) | |
| Severe | 13,492 (18,371) | | 10,131 (17,756) | | 6,684 (10,638) | | 5,330 (13,978) | | 31.8 (29.3; 34.3) | | 71.9% (60.6%; 84.1%) | |
| Psychiatric (DRG) |  | |  | |  | |  | |  | |  | |
| Mild | 5,153 (13,017) | | 4,657 (21,054) | | 2,184 (7,058) | | 1,482 (6,697) | | 90.0 (84.9; 95.3) | | 67.8% (57.7%; 78.5%) | |
| Moderate | 8,392 (84,752) | | 6,288 (30,881) | | 4,164 (8,357) | | 2,692 (8,433) | | 41.8 (39.3; 44.4) | | 63.6% (55.4%; 72.3%) | |
| Severe | 11,383 (27,124) | | 8,966 (18,068) | | 7,109 (20,446) | | 5,090 (38,959) | | 31.4 (28.9; 33.9) | | 72.0% (60.7%; 84.0%) | |
| Somatic |  | |  | |  | |  | |  | |  | |
| Mild | 5,790 (10,355) | | 3,450 (7,077) | | 6,050 (9,275) | | 3,573 (7,207) | | 0.7 (-1.3; 2.7) | | -2.4% (-7.2%; 2.6%) | |
| Moderate | 3,009 (6,270) | | 2,249 (5,137) | | 3,134 (6,534) | | 2,220 (5,461) | | 3.7 (1.3; 6.3) | | 0.9% (-4.2%; 6.2%) | |
| Severe | 2,939 (6,158) | | 1,896 (4,134) | | 3,247 (6,569) | | 2,144 (5,241) | | 3.1 (-0.3; 6.6) | | -6.2% (-12.6%; 0.6%) | |
| Somatic (DRG) |  | |  | |  | |  | |  | |  | |
| Mild | 8,102 (19,942) | | 4,517 (11,987) | | 8,691 (18,610) | | 4,923 (13,512) | | 0.6 (-1.4; 2.6) | | -4.5% (-10.0%; 1.4%) | |
| Moderate | 3,728 (10,579) | | 2,862 (9,192) | | 3,977 (11,525) | | 2,799 (8,787) | | 3.9 (1.4; 6.4) | | 1.2% (-5.0%; 7.7%) | |
| Severe | 3,710 (11,593) | | 2,355 (6,657) | | 4,334 (14,024) | | 2,903 (14,409) | | 3.0 (-0.4; 6.6) | | -5.2% (-12.9%; 3.2%) | |

^1^ The percentage change in risk of incurring any cost of that particular type of healthcare expenditure in the year after index date is given for TRD relative to matched non-TRD patients, presented with 95% confidence interval (CI).

^2^ The percentage change in costs in the year after the index date for TRD patients relative to matched non-TRD patients, presented with 95% confidence interval (CI).

^3^ For the period between 2006 to 2015, costs are presented based both on fixed prices and Diagnosis Related Groups (DRG) prices.

**S4 Table. Average healthcare resource utilization costs (EUR) in the year before and after the index date, stratified by year of MDD diagnosis**

|  | TRD | | Non-TRD | | TRD vs. non-TRD  (after index) | |
| --- | --- | --- | --- | --- | --- | --- |
| Variable | Before index  mean (SD) | After index  mean (sd) | Before index  mean (sd) | After index  mean (sd) | Change (%) in risk of cost  (95% CI)^1^ | Change (%) in costs (95% CI)^2^ |
| Total, excl. Medicine |  |  |  |  |  |  |
| 1996–2000 | 17,017 (20,867) | 8,885 (16,821) | 11,669 (16,009) | 7,545 (17,113) | 13.1 (11.2; 15.1) | 19.3% (11.9%; 27.3%) |
| 2001–2005 | 14,926 (19,256) | 10,119 (17,715) | 10,027 (13,948) | 6,507 (13,553) | 6.4 (5.5; 7.4) | 79.6% (70.4%; 89.4%) |
| 2006–2010 | 12,286 (16,480) | 9,418 (15,696) | 8,240 (11,116) | 6,023 (12,406) | 0.6 (0.5; 0.8) | 106.9% (98.2%; 116.0%) |
| 2011–2015 | 11,673 (15,474) | 9,172 (13,889) | 7,881 (10,299) | 5,656 (9,879) | 0.7 (0.5; 0.8) | 110.7% (101.8%; 120.0%) |
| Total, incl. Medicine |  |  |  |  |  |  |
| 1996–2000 | 17,801 (20,855) | 10,091 (16,889) | 12,254 (16,060) | 8,315 (17,186) | 2.9 (2.5; 3.3) | 73.3% (62.9%; 84.4%) |
| 2001–2005 | 15,724 (19,296) | 11,505 (17,904) | 10,649 (14,080) | 7,314 (13,719) | 1.8 (1.5; 2.0) | 107.3% (98.0%; 117.1%) |
| 2006–2010 | 12,957 (16,581) | 10,553 (15,915) | 8,785 (11,295) | 6,690 (12,625) | 0.5 (0.4; 0.6) | 105.8% (98.3%; 113.6%) |
| 2011–2015 | 12,172 (15,550) | 9,923 (14,049) | 8,287 (10,455) | 6,100 (10,048) | 0.5 (0.4; 0.7) | 109.3% (101.3%; 117.7%) |
| Psychiatric |  |  |  |  |  |  |
| 1996–2000 | 12,947 (20,028) | 6,493 (15,966) | 6,911 (13,900) | 4,711 (15,529) | 49.5 (44.8; 54.4) | 20.4% (11.1%; 30.5%) |
| 2001–2005 | 10,573 (17,774) | 7,466 (16,851) | 5,085 (11,457) | 3,532 (12,029) | 63.3 (59.5; 67.3) | 48.5% (39.3%; 58.2%) |
| 2006–2010 | 7,749 (14,604) | 6,466 (14,609) | 3,511 (8,283) | 3,004 (10,720) | 57.7 (54.9; 60.6) | 62.2% (54.4%; 70.5%) |
| 2011–2015 | 7,217 (12,814) | 6,161 (12,259) | 3,341 (7,056) | 2,620 (7,680) | 48.6 (46.1; 51.1) | 70.9% (63.3%; 78.9%) |
| Somatic |  |  |  |  |  |  |
| 1996–2000 | 4,070 (7,171) | 2,392 (5,141) | 4,758 (8,829) | 2,834 (7,116) | 0.8 (-2.6; 4.3) | -8.4% (-14.9%; -1.5%) |
| 2001–2005 | 4,278 (8,510) | 2,535 (5,582) | 4,885 (8,996) | 2,875 (6,409) | 1.0 (-1.3; 3.4) | -5.6% (-10.4%; -0.5%) |
| 2006–2010 | 4,136 (7,992) | 2,623 (5,533) | 4,409 (7,884) | 2,736 (6,221) | 2.0 (0.0; 4.0) | -0.2% (-4.6%; 4.3%) |
| 2011–2015 | 4,041 (8,483) | 2,675 (6,179) | 4,195 (7,842) | 2,738 (6,186) | 2.6 (0.6; 4.7) | -4.0% (-8.4%; 0.6%) |
| GP^3^ |  |  |  |  |  |  |
| 2006–2010 | 298 (216) | 229 (207) | 227 (183) | 182 (166) | 1.5 (1.2; 1.9) | 28.0% (24.9%; 31.3%) |
| 2011–2015 | 302 (282) | 223 (193) | 238 (182) | 186 (183) | 1.2 (0.8; 1.6) | 22.2% (19.0%; 25.4%) |
| Other private healthcare specialist^3^ | |  |  |  |  |  |
| 2006–2010 | 103 (204) | 100 (241) | 93 (221) | 101 (279) | 4.6 (3.0; 6.1) | 1.8% (-1.7%; 5.4%) |
| 2011–2015 | 114 (258) | 113 (296) | 107 (260) | 111 (324) | 4.1 (2.4; 5.9) | 2.5% (-1.4%; 6.5%) |
| Medicine, other |  |  |  |  |  |  |
| 1996–2000 | 343 (677) | 400 (770) | 291 (574) | 328 (648) | 6.3 (4.6; 8.1) | 21.0% (13.8%; 28.8%) |
| 2001–2005 | 384 (778) | 458 (871) | 347 (690) | 398 (792) | 5.0 (3.8; 6.3) | 24.3% (18.3%; 30.6%) |
| 2006–2010 | 371 (772) | 431 (885) | 344 (812) | 367 (846) | 6.7 (5.5; 7.8) | 24.4% (19.2%; 29.9%) |
| 2011–2015 | 305 (680) | 347 (691) | 282 (635) | 297 (677) | 6.7 (5.4; 7.9) | 22.5% (17.1%; 28.1%) |
| Medicine, other psychiatric | |  |  |  |  |  |
| 1996–2000 | 77 (134) | 85 (159) | 48 (93) | 47 (113) | 42.0 (37.9; 46.2) | 38.4% (30.5%; 46.9%) |
| 2001–2005 | 46 (104) | 52 (128) | 30 (76) | 33 (120) | 48.7 (44.6; 52.8) | 27.2% (20.7%; 34.1%) |
| 2006–2010 | 28 (78) | 33 (122) | 18 (68) | 20 (107) | 57.8 (52.9; 62.9) | 18.3% (12.2%; 24.8%) |
| 2011–2015 | 28 (71) | 35 (97) | 19 (66) | 19 (78) | 71.2 (64.7; 77.9) | 19.6% (12.7%; 27.0%) |
| Medicine, antidepressant | |  |  |  |  |  |
| 1996–2000 | 364 (309) | 721 (554) | 245 (293) | 394 (440) | 34.5 (32.7; 36.3) | 46.7% (40.7%; 52.9%) |
| 2001–2005 | 368 (419) | 877 (852) | 246 (366) | 376 (573) | 29.4 (28.1; 30.7) | 115.2% (107.1%; 123.7%) |
| 2006–2010 | 273 (432) | 671 (889) | 182 (396) | 280 (576) | 29.9 (28.8; 31.1) | 154.3% (143.8%; 165.2%) |
| 2011–2015 | 165 (292) | 369 (528) | 105 (264) | 129 (328) | 35.3 (34.0; 36.7) | 173.5% (162.8%; 184.5%) |

^1^ The percentage change in risk of incurring any cost of that particular type of healthcare expenditure in the year after index date is given for TRD relative to matched non-TRD patients, presented with 95% confidence interval (CI).

^2^ The percentage change in costs in the year after the index date for TRD patients relative to matched non-TRD patients, presented with 95% confidence interval (CI).

^3^ Data on GP and other private healthcare specialists not available before 2006.

**S5 Table. Average healthcare resource utilization comparing TRD patients with non-TRD patients in the matched population, abstaining from restriction to patients being alive 2 years after MDD index**

|  | TRD | | Non-TRD | TRD vs. non-TRD | | | | | | | |  |  |
| --- | --- | --- | --- | --- | --- | --- | --- | --- | --- | --- | --- | --- | --- |
| Variable | Mean (sd)^1^ | | Mean (sd)^1^ | Change (%) in risk  of utilization  (95% CI)^2^ | | p-value | Change (%) in  utilization (95% CI)^3^ | | | p-value |  |  |  |
| **Psychiatric contacts** | | |  |  | |  |  | | |  | | |  |
| Hospitalization | 0.3 (1.0) | | 0.1 (0.6) | 133.5 (125.2;142.1) | | <.0001 | 6.1 (3.9; 8.4) | | | <.0001 |  |  |  |
| Acute | 0.3 (0.9) | | 0.1 (0.6) | 131.1 (122.5;140.1) | | <.0001 | 5.4 (3.1; 7.7) | | | <.0001 |  |  |  |
| Elective | 0.0 (0.3) | | 0.0 (0.1) | 176.7 (150.7;205.3) | | <.0001 | 2.4 (-1.2; 6.1) | | | 0.19 |  |  |  |
| Hospital bed days | 10.1 (30.9) | | 5.0 (23.9) | 116.2 (110.1;122.4) | | <.0001 | -22.9 (-26.7;-19.0) | | | <.0001 |  |  |  |
| Acute | 8.5 (27.4) | | 4.1 (20.4) | 113.6 (107.3;120.1) | | <.0001 | -19.8 (-23.8;-15.6) | | | <.0001 |  |  |  |
| Elective | 1.6 (12.4) | | 0.9 (10.9) | 154.3 (134.6;175.8) | | <.0001 | -42.4 (-49.1;-34.9) | | | <.0001 |  |  |  |
| ED visit | 0.3 (1.4) | | 0.2 (0.8) | 85.8 (78.6; 93.4) | | <.0001 | 3.2 (0.4; 6.0) | | | 0.02 |  |  |  |
| Outpatient visit | 9.4 (16.6) | | 4.7 (12.5) | 65.8 (63.7; 67.9) | | <.0001 | 31.6 (28.8; 34.5) | | | <.0001 |  |  |  |
| Home visit | 1.9 (7.6) | | 0.7 (4.1) | 129.9 (120.9;139.3) | | <.0001 | 27.4 (21.8; 33.2) | | | <.0001 |  |  |  |
| Private psychiatrist visit | 0.8 (2.3) | | 0.3 (1.3) | 129.7 (120.3;139.5) | | <.0001 | 25.0 (20.7; 29.4) | | | <.0001 |  |  |  |
| Private psychologist visit | 0.2 (1.1) | | 0.2 (0.9) | 35.3 (27.8; 43.2) | | <.0001 | -0.5 (-4.8; 4.0) | | | 0.81 |  |  |  |
| **Somatic contacts** | |  | | |  | | |  |  | | | | |
| Hospitalization | 0.7 (1.7) | | 0.6 (1.7) | 6.4 (4.2; 8.7) | | <.0001 | 0.6 (-1.1; 2.4) | | | 0.46 |  |  |  |
| Acute | 0.5 (1.5) | | 0.5 (1.3) | 8.8 (6.3; 11.5) | | <.0001 | 0.5 (-1.3; 2.3) | | | 0.58 |  |  |  |
| Elective | 0.1 (0.5) | | 0.1 (0.8) | -0.5 (-4.6; 3.8) | | 0.83 | -1.1 (-3.0; 0.9) | | | 0.29 |  |  |  |
| Hospital bed days | 3.7 (11.9) | | 4.3 (14.4) | 4.4 (2.2; 6.5) | | <.0001 | -14.6 (-17.1;-12.0) | | | <.0001 |  |  |  |
| Acute | 2.8 (9.6) | | 3.1 (10.8) | 7.0 (4.5; 9.5) | | <.0001 | -13.1 (-15.7;-10.3) | | | <.0001 |  |  |  |
| Elective | 0.9 (5.2) | | 1.2 (7.4) | -4.3 (-8.2; -0.3) | | 0.03 | -18.9 (-23.0;-14.6) | | | <.0001 |  |  |  |
| ED visit | 0.4 (1.1) | | 0.4 (1.1) | 2.3 (-0.2; 4.9) | | 0.07 | -0.5 (-2.0; 1.0) | | | 0.48 |  |  |  |
| Outpatient visit | 3.1 (7.3) | | 3.2 (8.6) | 7.7 (6.3; 9.1) | | <.0001 | -4.1 (-5.9; -2.3) | | | <.0001 |  |  |  |
| **GP visit** | 9.1 (9.3) | | 7.2 (8.2) | 5.8 (5.2; 6.5) | | <.0001 | 21.6 (20.0; 23.2) | | | <.0001 |  |  |  |
| **Other healthcare specialist visit** | 1.9 (3.1) | | 1.6 (2.9) | 8.2 (7.0; 9.4) | | <.0001 | 3.6 (2.1; 5.2) | | | <.0001 |  |  |  |

The present analysis did not apply restrictions on being alive, not having migrated and not having acquired diagnoses for other psychiatric disorders 2 years after MDD index date.

^1^ Mean healthcare resource utilization with standard deviation (sd) in the year following the index date.

^2^ The percentage change in risk of having any utilization of the particular healthcare service in the year after the index date is given for TRD relative to matched non-TRD patients, presented with 95% confidence interval (CI).

^3^ The percentage change in utilization in the year after the index date for TRD patients relative to matched non-TRD patients, among patients who have some healthcare resource utilization.
