# Supplementary material for: Healthcare resource utilization in patients with treatment-resistant depression—A Danish national registry study
Source: PLoS One. 2022 Sep 27;17(9):e0275299. doi: 10.1371/journal.pone.0275299 (PMC9514626; doi:10.1371/journal.pone.0275299)
Supplement: Attachment — Submitted filename: Rebuttal letter.docx [file pone.0275299.s002.docx]

Copenhagen, 12^th^ August 2022

Rebuttal letter, PlosOne PONE-D-22-09950

Thank you for reviewing our work and for your comments and suggestions. Please find below our point-to-point address of the items raised by the editor and reviewers. We hope you will find our responses and revisions satisfactory.

On behalf of the author team,

Kind regards

Kristoffer Jarlov Jensen

1. Please ensure that your manuscript meets PLOS ONE's style requirements, including those for file naming. The PLOS ONE style templates can be found at

https://journals.plos.org/plosone/s/file?id=wjVg/PLOSOne_formatting_sample_main_body.pdf and

https://journals.plos.org/plosone/s/file?id=ba62/PLOSOne_formatting_sample_title_authors_affiliations.pdf

**Author’s reply:**

**We have amended the format according the PLOS ONE's style requirements.**

2. Please provide additional details regarding participant consent. In the ethics statement in the Methods and online submission information, please ensure that you have specified (1) whether consent was informed and (2) what type you obtained (for instance, written or verbal, and if verbal, how it was documented and witnessed). If your study included minors, state whether you obtained consent from parents or guardians. If the need for consent was waived by the ethics committee, please include this information.

If you are reporting a retrospective study of medical records or archived samples, please ensure that you have discussed whether all data were fully anonymized before you accessed them and/or whether the IRB or ethics committee waived the requirement for informed consent. If patients provided informed written consent to have data from their medical records used in research, please include this information.

**Author’s reply: We have added to the ethics section that data was pseudonymized and informed consent was not required according to Danish law.**

3. We note that you have included the phrase “data not shown” in your manuscript. Unfortunately, this does not meet our data sharing requirements. PLOS does not permit references to inaccessible data. We require that authors provide all relevant data within the paper, Supporting Information files, or in an acceptable, public repository. Please add a citation to support this phrase or upload the data that corresponds with these findings to a stable repository (such as Figshare or Dryad) and provide and URLs, DOIs, or accession numbers that may be used to access these data. Or, if the data are not a core part of the research being presented in your study, we ask that you remove the phrase that refers to these data.

**Author’s reply: The results of the sensitivity analyses previously referred to as “data not shown” s now found in the Supporting Material (S5 Table).**

4. Please include captions for your Supporting Information files at the end of your manuscript, and update any in-text citations to match accordingly. Please see our Supporting Information guidelines for more information: http://journals.plos.org/plosone/s/supporting-information.

**Author’s reply: We have amended the manuscript and Supporting material accordingly.**

5. In your Data Availability statement, you have not specified where the minimal data set underlying the results described in your manuscript can be found. PLOS defines a study's minimal data set as the underlying data used to reach the conclusions drawn in the manuscript and any additional data required to replicate the reported study findings in their entirety. All PLOS journals require that the minimal data set be made fully available. For more information about our data policy, please see http://journals.plos.org/plosone/s/data-availability.

Upon re-submitting your revised manuscript, please upload your study’s minimal underlying data set as either Supporting Information files or to a stable, public repository and include the relevant URLs, DOIs, or accession numbers within your revised cover letter. For a list of acceptable repositories, please see http://journals.plos.org/plosone/s/data-availability#loc-recommended-repositories. Any potentially identifying patient information must be fully anonymized.

Important: If there are ethical or legal restrictions to sharing your data publicly, please explain these restrictions in detail. Please see our guidelines for more information on what we consider unacceptable restrictions to publicly sharing data: http://journals.plos.org/plosone/s/data-availability#loc-unacceptable-data-access-restrictions. Note that it is not acceptable for the authors to be the sole named individuals responsible for ensuring data access.

We will update your Data Availability statement to reflect the information you provide in your cover letter.

**Author’s reply: We have added to the Data Availability statement (found at the end of the manuscript) that in order to get access to the data underlying the present results, applications must be sent to the relevant government bodies in Denmark, the Danish Data Protection Agency and the data owners, Statistics Denmark and the Danish Health Data Authority, as stipulated by Danish law.**

6. Thank you for stating the following financial disclosure:

“The study was financed by Janssen Cilag A/S.”

Please state what role the funders took in the study. If the funders had no role, please state: "The funders had no role in study design, data collection and analysis, decision to publish, or preparation of the manuscript."

If this statement is not correct you must amend it as needed.

Please include this amended Role of Funder statement in your cover letter; we will change the online submission form on your behalf.

**Author’s reply: We have added the following to the cover letter: “The funders contributed to conception of the study and writing of the manuscript.”**

7. Thank you for stating the following in the Competing Interests section:

“The study was performed by the Copenhagen Phase IV Unit (Phase4CPH) and was financed by Janssen Cilag A/S, which holds license to antidepressant medicine. Via Phase4CPH, JP, EJS and MZA have performed other studies regarding antidepressants involving funding from Janssen Cilag and Eli Lilly, while KJJ, FHG and MO have performed other studies regarding antidepressants involving funding from Janssen Cilag. All funds were given to their institution. JR, NB and SA are employees of Janssen Cilag. Janssen Cilag A/S and its employees did not have access to the registry data located on the servers of Statistics Denmark and had therefore no influence on the data management and data analysis of the present work. The authors at Copenhagen Phase IV Unit retained editorial control of the manuscript.”

Please confirm that this does not alter your adherence to all PLOS ONE policies on sharing data and materials, by including the following statement: "This does not alter our adherence to PLOS ONE policies on sharing data and materials.” (as detailed online in our guide for authors http://journals.plos.org/plosone/s/competing-interests). If there are restrictions on sharing of data and/or materials, please state these. Please note that we cannot proceed with consideration of your article until this information has been declared.

Please include your updated Competing Interests statement in your cover letter; we will change the online submission form on your behalf.

**Author’s reply: We have amended the Competing Interests statement accordingly.**

8. Please review your reference list to ensure that it is complete and correct. If you have cited papers that have been retracted, please include the rationale for doing so in the manuscript text, or remove these references and replace them with relevant current references. Any changes to the reference list should be mentioned in the rebuttal letter that accompanies your revised manuscript. If you need to cite a retracted article, indicate the article’s retracted status in the References list and also include a citation and full reference for the retraction notice.

**Author’s reply: We believe the reference list is correct and does not contain any retracted papers.**

Reviewers' comments:

Reviewer's Responses to Questions

Comments to the Author

1. Is the manuscript technically sound, and do the data support the conclusions?

The manuscript must describe a technically sound piece of scientific research with data that supports the conclusions. Experiments must have been conducted rigorously, with appropriate controls, replication, and sample sizes. The conclusions must be drawn appropriately based on the data presented.

Reviewer #1: Yes

Reviewer #2: Yes

________________________________________

2. Has the statistical analysis been performed appropriately and rigorously?

Reviewer #1: Yes

Reviewer #2: Yes

________________________________________

3. Have the authors made all data underlying the findings in their manuscript fully available?

The PLOS Data policy requires authors to make all data underlying the findings described in their manuscript fully available without restriction, with rare exception (please refer to the Data Availability Statement in the manuscript PDF file). The data should be provided as part of the manuscript or its supporting information, or deposited to a public repository. For example, in addition to summary statistics, the data points behind means, medians and variance measures should be available. If there are restrictions on publicly sharing data—e.g. participant privacy or use of data from a third party—those must be specified.

Reviewer #1: Yes

Reviewer #2: Yes

________________________________________

4. Is the manuscript presented in an intelligible fashion and written in standard English?

PLOS ONE does not copyedit accepted manuscripts, so the language in submitted articles must be clear, correct, and unambiguous. Any typographical or grammatical errors should be corrected at revision, so please note any specific errors here.

Reviewer #1: Yes

Reviewer #2: Yes

________________________________________

5. Review Comments to the Author

Please use the space provided to explain your answers to the questions above. You may also include additional comments for the author, including concerns about dual publication, research ethics, or publication ethics. (Please upload your review as an attachment if it exceeds 20,000 characters)

Reviewer #1: The rationale for the lack of some data only from 2006 onwards should be mentioned and the consequences of the lack of data should be explained. The lack of data should be included in the weaknesses of the research

**Author’s reply: This has now been addressed in the Methods section and the Strengths and weaknesses section.**

In the section “depression severity”, it is difficult to understand the meaning of mild depression as a more heterogeneous group, the argument on anti-depressive treatment should be more elaborated

**Author’s reply:**

**We have attempted to explain this more clearly in the manuscript. In brief, the arguments are the following:**

1. **A central criterion for entering the study is hospital contact with depression.**
2. **Mild depression is often treated in primary care and not in hospital settings. Hence, the study participants with mild depression are in terms of psychiatric condition somewhat more “exceptional” or heterogenous compared to patients with severe depression, putatively having with a wider spread of utilization frequencies.**
3. **Therefore, having two shifts in antidepressant treatment will act as a stronger discriminator of psychiatric treatment need in this heterogenous group, compared with the patients with severe depression, whom already overall (on average) have a relatively strong psychiatric need.**

In the section “Comparison with other studies”, concurrent somatic comorbidities should be better explained, and the contradictions with the existing study should be further explored, the explanation is not sufficient.

**Author’s reply:**

**We have elaborated on the section discussing the literature on the association between TRD and somatic disease, including one more reference study. We hope the reviewer finds our revisions clarifying.**

Reviewer #2: Dear, I reviewed "Healthcare resource utilization in patients with treatment-resistant depression – a

Danish national registry study". thank you for your working. it's well written and methodologically corrected
